# Standardizing assessment practices of undergraduate medical competencies across medical schools: challenges, opportunities and lessons learned from a consortium of medical schools in Uganda

**DOI:** 10.11604/pamj.2014.19.382.5283

**Published:** 2014-12-16

**Authors:** Aloysius Gonzaga Mubuuke, Catherine Mwesigwa, Samuel Maling, Godfrey Rukundo, Mike Kagawa, David Lagoro Kitara, Sarah Kiguli

**Affiliations:** 1 Makerere University, College of Health Sciences, Kampala, Uganda; 2 Mbarara University of Science & Technology, Faculty of Medicine, Mbarara, Uganda; 3 Kampala International University, Faculty of Medicine, Kampala, Uganda; 4 Gulu University, Faculty of Medicine, Gulu, Uganda

**Keywords:** MESAU, competencies, assessment

## Abstract

**Introduction:**

Health professions education is gradually moving away from the more traditional approaches to new innovative ways of training aimed at producing professionals with the necessary competencies to address the community health needs. In response to these emerging trends, Medical Education for Equitable Services to All Ugandans (MESAU), a consortium of Ugandan medical schools developed key competencies desirable of graduates and successfully implemented Competency Based Education (CBE) for undergraduate medical students.

**Objectives:**

To examine the current situation and establish whether assessment methods of the competencies are standardized across MESAU schools as well as establish the challenges, opportunities and lessons learned from the MESAU consortium.

**Methods:**

It was a cross-sectional descriptive study involving faculty of the medical schools in Uganda. Data was collected using focus group discussions and document reviews. Findings were presented in form of themes.

**Results:**

Although the MESAU schools have implemented the developed competencies within their curricular, the assessment methods are still not standardized with each institution having its own assessment procedures. Lack of knowledge and skills regarding assessment of the competencies was evident amongst the faculty. The fear for change amongst lecturers was also noted as a major challenge. However, the institutional collaboration created while developing competencies was identified as key strength.

**Conclusion:**

Findings demonstrated that despite having common competencies, there is no standardized assessment blue print applicable to all MESAU schools. Continued collaboration and faculty development in assessment is strongly recommended.

## Introduction

There has been a global trend in health professions education to move away from the more traditional teacher-centred learning to more innovative student-centred learning approaches such as problem based learning and competency based education [1, 2]. Such changes in teaching and learning also require changes in student assessment in order to achieve an effective constructive alignment [3, 4]. It has been reported that effective student assessment drives learning and that assessment plays a crucial role in the learning process [5, 6]. Literature is replete with documentation about student assessment in health professions education.

Davis and Harden [7] describe assessment as the single most important determinant of what students actually learn (as opposed to what they are taught) and is considered to be uniquely powerful as a tool for manipulating the whole education process. Epstein and Huder [8] further supplement that assessment becomes the most important basis of the medical education system to public accountability. In order to achieve maximum benefits from assessment activities, it should not be detached from the planning of the curriculum and should always be an active component in curriculum planning activities [9, 10].

Van der Vleuten [11] describes assessment as an evaluation of student performance against set learning outcomes and Bridge et al [12] further supplement that the basic tenets of any assessment are 1) validity (whether the chosen method measures what it claims to measure), 2) reliability (the degree to which the measurement is accurate and reproducible), 3) acceptability (the extent to which stakeholders; students, faculty and patients, endorse the measure and associated interpretation of the scores), 4) feasibility (affordability and efficiency of the assessment method for testing purposes) and 5) educational impact (the capacity of the assessment method to drive students’ study efforts based on the curriculum requirements and how assessment leads to overall improvement on the curriculum.

The choice of any assessment method depends on the purpose of the assessment and the student competencies being assessed at any one particular time [13]. Thus different student competencies need to use different assessment methods in order to make objective judgments about student performance. It is thus evident from previous literature that suitable assessment methods need to be in place in order to assist lecturers make objective judgments about students.

Innovations in medical education have led to the emergency of what is called transformative learning where teaching and learning no longer focus on knowledge only, but also emphasize other professional competencies such as clinical/practical skills, communication skills, leadership, professionalism and ethical practice [7]. Such type of learning is likely to produce medical professionals who are well grounded in not only knowledge, but also in other graduate attributes that make a complete professional and which are necessary to meet today`s health needs of the population. In such learning, student competencies have to be clearly defined and assessment designed to evaluate those competencies [10].

In order to address the then challenges in health professions education in Uganda and produce professionals with the desirable competencies to address the community health needs, five Ugandan medical schools teamed up to form a consortium called Medical Education for Services to All Ugandans (MESAU) under the auspices of Medical Education Partnership Initiative (MEPI) with a task of commencing CBE amongst the five schools. These schools included: Makerere University College of Health Sciences (MaKCHS), Mbarara University of Science and Technology medical school, Gulu University medical school, Kampala International University medical school and Busitema University medical school. Through wide consultations with stakeholders and a series of meetings, workshops and seminars, the MESAU consortium came up with nine key competency domains for all the five medical schools to be incorporated into the various medical school curricula (Table 1). This was followed by curricular reviews across all the medical schools to incorporate the competencies into the undergraduate teaching.

**Table 1:** The nine MESAU competencies

	Domain of Competencies
1.	Medical Knowledge
2.	Clinical skills
3.	Critical Inquiry and scientific method
4.	Professionalism
5.	Interpersonal and Communication skills
6.	Leadership and Management skills
7.	Population Health
8.	Continuous Improvement of Care through Reflective Practice
9.	Health Systems Management

Having adopted common competencies across the medical schools, with an aim of producing graduates with common attributes, the assessment of such competencies also ought to become standard and uniform across the medical schools. However, it is not known as to whether the assessment of the competencies is standard across MESAU schools. Focusing on four key competencies of *Medical knowledge, Clinical Skills, Professionalism and Communication skills*, the purpose of this study was to examine the current situation and establish whether assessment practices of the competencies were similar across the MESAU schools. Additionally, the study aimed at establishing the challenges faced, opportunities therein and lessons learned regarding the assessment of the aforementioned competencies.

## Methods

***Research design* :** it was a cross-sectional qualitative study investigating the assessment of undergraduate competencies amongst the MESAU institutions of Makerere University College of Health Sciences, Mbarara University of Science and Technology, Gulu University, Busitema University and Kampala International University. The study focused on four competencies of the medical curriculum namely; *Medical Knowledge, Clinical Skills, Professionalism and Communication Skills*.

*Study participants* : the study included lecturers who were selected purposively. Only those lecturers involved in teaching and assessment of competencies within the undergraduate medical curriculum were chosen.

***Data collection* :** data was collected using two methods. Initially, five focus group discussions were conducted, one focus group from each MESAU institution. Each focus group had 6 lecturers. Subsequently, document review of the medical curricular documents was done specifically checking for assessment methods of the competences. Responses from focus group discussions were audio-recorded and later transcribed. Information from the curricular documents was abstracted into a coding sheet.

***Data analysis* :** thematic analysis was used. Raw data was read and through a series of iterative and inductive open and axial coding, codes and themes were developed manually.

***Quality assurance*:** data was stored electronically and secured with a password. Participants were invited to validate interview transcripts and the emerging themes as well. Additionally, researcher bias was minimized by the researchers avoiding all pre-conceived ideas or experiences on the subject under investigation and practicing reflexivity and bracketing throughout the research process.

***Ethical considerations*:** participants provided written informed consent. Additionally, they were not identified by name and their responses were kept anonymous and confidential. Permission to conduct this study was granted by the Research and Ethics Committee, School of Health Sciences, College of Health Sciences, Makerere University.

## Results

The findings from this study are summarized under the following themes

### Assessment of the competencies: the current situation

Analysis of data showed that the MESAU schools were indeed implementing competency based education. The competencies of *Medical knowledge, Clinical skills, Professionalism and Communication skills* were well documented and faculty were aware of these competencies as developed by the MESAU consortium. Regarding the assessment of these competencies, findings from the data showed significant variations across the MESAU schools. Table 2 illustrates the assessment methods of each competency within each medical school. From the table, all the medical schools seem to concentrate on assessing knowledge and skills. Majority of the schools lack properly documented methods for assessing soft skills like professionalism and communication. Additionally, there is clear evidence that assessment methods are generally not the same across the medical schools despite the fact that they are assessing the same competencies. Each medical school seems to be using its own assessment methods. During the interviews and focus group discussions, this clearly came out as illustrated in the response below:

**Table 2:** Showing the assessment methods of the medical competencies across the MESAU Schools

MESAU Medical School	Assessment of Medical Knowledge	Assessment of Clinical Skills	Assessment of Professionalism	Assessment of Communication Skills
Makerere University College of Health Sciences	Multiple Choice Questions (MCQs), Extended Matching Items (EMIs), Structured questions, Short answer questions, Long essays and Tutorial based/small group assessment	Objective Structured Clinical Exam (OSCE), Mini-Clinical Exams, Simulated Patients in Skills Lab	Tutorial based/Small group assessment, Student Portfolios	Tutorial based/Small group assessment, OSCE stations on communication, Direct Observation, Simulations in Skills Lab
Mbarara University of Science and Technology	Multiple Choice Questions (MCQs), Long essays and Structured questions, Oral exams	Objective Structured Clinical Exam (OSCE	-	-
Gulu University	Multiple Choice Questions (MCQs), Long essays and Structured questions, Oral exams	Objective Structured Clinical Exam (OSCE	-	-
Kampala International University	Multiple Choice Questions (MCQs), Long essays and, Oral examsStructured questions	Objective Structured Clinical Exam (OSCE	-	-
Busitema University	Multiple Choice Questions (MCQs), Long essays and Structured questions	-	-	-

*``Although we got some training and workshops on CBE together with other medical schools in the consortium, I do not remember discussing and adopting common assessment methods for these competencies. We just devised our own within our individual school``*.

### Challenges

Another major theme that emerged from the data pointed at challenges faced as far as assessment of competencies was concerned. One key challenge that was prominent in the data gathered was a lack of a standard and uniform assessment plan and blue print that seemed to be overlooked during the development of the competencies. *``While we gathered together as a consortium of medical schools to develop common competencies, we unfortunately overlooked the idea of simultaneously adopting common assessment methods for each competency``*.

*``Since we want to produce graduates with similar attributes for our population needs, we must have curricular with similar attributes across all the medical schools which we did effectively during stakeholders meetings. However, we must also have similar assessment methods for those attributes which we unfortunately gave little attention``*.

From the above responses, it can be observed that while common competencies desirable of graduates were adopted for the MESAU schools, assessment methods that should be uniform and standard across the schools seemed to have received less attention during the process of developing competencies.

Another key challenge identified was limited knowledge of lecturers regarding the assessment of competencies. There seemed to be a knowledge gap amongst the lecturers as to how to assess the new competencies and they seemed to be deficient of the suitable assessment methods for each competency. The following response reflects this general observation:

*``To many of us, this competency based education is a new thing. We do not know how to effectively assess students in many students. Even the competencies where we have some experience like medical knowledge, there are still issues. To make matters worse, each medical school seems to be assessing the competencies using their own methods``*.

The issue of higher education set up in Uganda where each tertiary institution is independent defining their own way of doing things was another challenge observed. Almost all participants cited the unfavorable set up within the higher education sector. This is reflected in the response below:

*``We do not have common examining boards for higher institutions of learning including medical schools. As a result it is difficult to adopt very similar assessment practices across all medical schools. Each medical school may want to safe guard their own methods and this is still a challenge``*.

The fear for change was arguably the biggest challenge. Many participants in this study expressed concerns about adopting common assessment practices for the newly developed competencies that target moving away from the traditional methods of assessing students. For example, many lecturers believed that one would assess communication through oral exams. Still many were afraid of adopting new methods of doing things perhaps due to fear of moving away from the comfort zone. The following response was typical.

*``Am used to the way we have been assessing students without these competencies. I appreciate the introduction of the competencies, but I hope if we do have common assessment practices, we shall remain with authority as lecturers``*.

From the response above, one can observe that there is fear that lecturers will lose their authority of assessing students the way it has always been done if common assessment practices are adopted across the medical schools.

### Opportunities

However, there were also some opportunities cited. Having developed and adopted the medical competencies across the MESAU schools, this provides an opportunity to collaborate across the medical schools to develop common assessment methods for each of the competencies in future. The momentum gathered during the development of the competencies can be extended to developing assessment methods. This opportunity was reflected in the response below:

*``Since we can now collaborate as medical schools, this provides us a chance to have similar assessment methods for the competencies such that we can use the same benchmarks when assessing students``*.

Another opportunity that emerged from the data was the possibility of having joint planning meetings since the medical schools can now communicate to each other within the consortium.

*``The idea of being in a consortium as medical schools gives us an opportunity to organize assessment planning meetings. This will give us a chance to have standard assessment methods that are similar across the medical schools``*.

Having a unified voice for the Ugandan medical schools to advocate for change in policies governing higher institutions of learning was another opportunity. *``We could use our voice and strength as a consortium to advocate for change whereby we can suggest the possibility of having common assessment boards for medical schools``*, said one participant.

Lastly, there was an opportunity within the consortium of sharing best assessment practices as some of the schools seemed to be a head of others.

### Lessons learned and way forward

The participants in this study also highlighted key lessons and a way forward. One key lesson was the benefit of collaboration and team work across the medical schools. Participants observed that the consortium approach had enhanced collaboration across the medical schools which was not the case before and this collaboration could be used as a foundation to advocate for standardized assessment practices across the schools. Subsequently, it was suggested that having joint faculty development programmes in assessment is essential as many participants felt they had less knowledge and skills in assessing the new competencies.

*``The major solution is faculty development. Many of us are ignorant about assessment of competencies and we need to have joint training programmes across all the medical school…``*

Another key lesson learned was the practice of looking at training in its entirety not just focusing on few aspects. Participants felt that assessment methods should have been discussed alongside the competencies with all stakeholders, but this was not the case. Therefore, it is essential to scrutinize the curriculum as a whole including content, teaching and learning methods, assessment, implementation issues and evaluation plans.

Participants suggested going through the same procedure of identifying stakeholders such that assessment methods are developed collaboratively with involvement of all stakeholders like it was done for the competencies.

*``Since we came up with competencies as a team of stakeholders, let us also identify those stakeholders and develop assessment methods as a team``*.

Continuous engagement of medical schools across the consortium and sharing of best practices was also suggested as a way forward. This continuous engagement was also suggested as a way of continuously sensitizing faculty about the benefits of having standardized assessment practices across a consortium of medical schools that target having graduates with similar competencies.

*``We should continuously share best practices through seminars and training workshops as a way of improving and as a change management strategy because such continuous engagement can create buy-in from those lecturers that are afraid of change``*, one participant said.

## Discussion

The purpose of this study was to examine the current situation and establish whether assessment practices of the selected competencies of *Medical knowledge, Clinical Skills, Professionalism and Communication skills* were similar across the MESAU schools. Additionally, the study aimed at finding out the key challenges, opportunities that exist, lessons learned and way forward regarding assessment of competencies. Findings indicate that the various medical schools within the MESAU consortium have different assessment methods for the above competencies although the teaching of competencies has already commenced across the schools. This observation should be considered in light of the fact that these MESAU schools adopted common undergraduate medical competencies. In a situation where the medical schools collaboratively developed competencies involving all stakeholders, one would expect the schools to equally have similar assessment methods if health professionals with similar attributes are to be produced.

However, this was not the case. Although the medial schools have similar competencies, each school seems to have their own assessment methods. One can discern that if assessment of the competencies is not standardized across the medical schools, the graduates are likely to come out with different attributes, defeating the original intention of developing common competencies within a consortium. The most plausible explanation for this goes to the way higher education is set up in Uganda where institutions are autonomous in what they do with limited opportunities of teaming up. This was observed as a major challenge to developing common assessment practices across the consortium.

What majorly compounds this challenge of lacking common assessment methods is what can be called *institutionalism*. All the medical schools are somewhat autonomous, each school defining their own agenda and each school trying to protect their own interests. As a result, the medical schools have been engaging in some form of competition to be the best in the country. In this situation, medical schools are less likely to come together to share best practices and collaboratively map a way forward. This unfortunately extends into student assessment where each school would not like to share what they use. This possibly explains as to why assessment of competencies is still not standard across the schools. However, the MESAU consortium has tried to break through institutional barriers and there has been significant collaboration across the medical schools. This provides a firm basis on to which collaboratively developing assessment plans can take place.

From the findings, most of the medical schools still emphasize knowledge and skills and pay limited attention to soft skills like professionalism and communication, an observation that resonates well with previous literature [9, 10]. Some of these soft skills were never emphasized in the traditional medical curricular and lecturers paid little attention to them. This coupled with the fact that lecturers never received training in how to assess soft skills possibly explains the observation that such skills are not as prominent as other practical skills. Makerere University College of Health Sciences is the oldest institution in Uganda and has been the leader of health professions education for some time. This could offer an explanation as to why the institution has devised some measures to assess soft skills like professionalism and communication. On the contrary, Busitema University Medical School has recently just been approved to commence training which could possibly explain their lack of assessment methods for clinical skills, professionalism and communication since there are no students in clinical years as yet.

Inadequate knowledge and skills of faculty on how to assess the various competencies was identified as a major challenge. Training faculty in CBE including assessment has been emphasized in previous literature [7]. In their study, Davis and Harden [7] reported that having well trained faculty with knowledge of CBE and how to objectively assess the various competencies is key to successfully implement CBE. In this study, lecturers had limited expertise in assessing the newly developed competencies partly because there had been no faculty development programmes prior to commencing CBE. It appears like many of the lecturers still depend on the traditional assessment methods to assess the competencies. Unfortunately, many of these assessment methods may not be valid and reliable in assessing the new competencies.

For example, using an oral exam to assess students` communication skills is questionable as it might not give an overall picture of how good or bad the student is at communication. With new competencies being emphasized in health professions education like communication, reflection, ethics and professionalism, new assessment methods for these competencies need to be developed putting into consideration the resources available. Van der Vleuten and Schuwirth [13] caution that depending on traditional assessment methods to assess the newly emerging competencies is a disservice to the students. Therefore, the need to train faculty in assessment is paramount.

Similarly, the fear of change was a major factor noted in this study. Many lecturers seemed to be comfortable with the way they had been assessing students. Some of them felt that their authority would be taken away if common assessment methods were adopted. The fear to change from the traditional of teaching and assessing students has been reported previously [3]. Findings from this study are in resonance with the previous studies that faculty fear change. The reason as to why this could be so is the fear of the unknown. Many lecturers in medical schools are not education experts and many of these concepts and principles are new to them. To many they do not make sense.

This fear for change also seems to emanate from breaking of institutional boundaries. Under MESAU, the medical schools in Uganda teamed up and are supposed to collaborate without boundaries in order to achieve a common goal of producing graduates with similar attributes. However, many lecturers in individual schools appear to be apprehensive about this, fearing that their dominance and authority within their medical schools will be threatened. Unfortunately, this fear of change hampers acceptability of new innovations and instead breeds compliance rather than acceptance. Abandoning traditional assessment methods and adopting new innovative ones is thus a major challenge. Unfortunately, with the new common competencies that MESAU medical schools adopted as a consortium, there should be valid and reliable assessment methods of these competencies that are standardized across the medical schools if the original goal of the consortium is to be fully realized. Continuous engagement of the faculty across the schools explaining to them the benefits of the new innovations is thus needed.

A key opportunity that was identified in this study was the chance to collaborate and work in teams across the medical schools. The MESAU consortium provides a great opportunity for the medical schools to come together on a single platform and develop standardized assessment methods for the competences across the schools. This opportunity never existed before. If the ultimate goal is to have graduates with similar competencies that are needed to address health challenges in the community, the assessment of these competencies should therefore be similar across the schools. MESAU has nurtured the concept of team work and collaboration and there is need to continuously build on this. What is needed right now is the development of standardized assessment practices across the schools that are feasible and acceptable cognizant of the resources available.

Thus the major implication of this study on current practice is the idea of team work, constant collaboration and stakeholder involvement and engagement to improve teaching, learning and assessment in health professions education. It is only through collaboration and sharing best practices across the medical schools in Africa that the contribution to the reduction of the disease burden will be significant. Each individual school has strengths, weaknesses and opportunities. Working together enhances the strengths and minimizes the weaknesses while at the same time, creates more opportunities for us to improve not only training, but also ultimately, the health of the communities. Despite the challenges faced, the MESAU consortium in Uganda has tried to bring the medical schools together to initiate this process of collaboration and team work. Developing common competencies is itself a milestone and the medical schools that never used to communicate can now communicate with each other and speak with one unified voice to advocate for change.

This study also has some implications for future research. With the autonomy of higher institutions of learning in many countries where each institution develops its own curricular and designs its own assessment methods, the issue of standardizing assessment practices across all medical schools in a country needs further investigation. This is a key direction for future research in this area. Another direction for future research would be to investigate whether medical schools teaming up together to form consortia can have greater impact on advocating for change in policies that affect both training and health care provision.

In this study, non-probabilistic sampling was used to select participants, which is a limitation and thus findings may not be fully generalized to other settings. Additionally, the fact that not all lecturers in the medical schools were involved; some important information could have been missed from them which could probably have added richness to the findings, another limitation of the study. However, the study provides useful findings upon which more studies can build.

## Conclusion

The MESAU consortium successfully developed common competencies which have been successfully implemented within the undergraduate medical training across Ugandan medical schools. Despite this success however, there are still challenges of standardizing assessment methods of the competencies across the medical schools. The collaboration amongst the medical schools within the consortium however provides a useful platform upon which the institutions can come together to standardize assessment of the developed competencies. Such collaboration can also provide key lessons to other medical schools in Africa and beyond.
